# Normal Pressure Hydrocephalus Following Cranial Radiation: Identification of Shunting Responders

**DOI:** 10.3390/cancers15071949

**Published:** 2023-03-24

**Authors:** Nuria Cayuela, Manuel Domínguez-Lizarbe, Gerard Plans, Montserrat Alemany, Juan José Sánchez, Begoña Andrés, Anna Lucas, Jordi Bruna, Marta Simó

**Affiliations:** 1 Neurology Department, Complex Hospitalari Moisès Broggi, 08970 Sant Joan Despí, Barcelona, Spain; 2 Neurology Department, Hospital Germans Trias i Pujol, Universitat Autònoma de Barcelona, 08916 Badalona, Barcelona, Spain; 3 Neuro-Oncology Unit, Hospital Universitari de Bellvitge-Institut Català d’Oncologia l’Hospitalet, Institut d’Investigació Biomèdica de Bellvitge (Oncobell Program), 08908 L’Hospitalet de Llobregat, Barcelona, Spain; 4 Institute for Diagnostic Imaging, Hospital Universitari de Bellvitge, 08907 L’Hospitalet de Llobregat, Barcelona, Spain; 5 Radiation Oncology Department, Hospital Universitari de Bellvitge-Institut Català d’Oncologia l’Hospitalet, 08908 L’Hospitalet de Llobregat, Barcelona, Spain; 6 Cognition and Brain Plasticity Group, Institut d’Investigació Biomèdica de Bellvitge, 08908 L’Hospitalet de Llobregat, Barcelona, Spain

**Keywords:** cancer survivors, radiotherapy, normal pressure hydrocephalus, ventriculoperitoneal shunt, volumetric analysis

## Abstract

**Simple Summary:**

Up to 50–90% of long-term cancer survivors will exhibit moderate to severe cognitive impairment following cranial radiotherapy (RT). It is common in this population to observe a ventricular dilatation disproportionate to the cerebral atrophy, which clinically manifests similar to normal pressure hydrocephalus (NPH). Previous studies demonstrated that early placement of a ventriculoperitoneal shunt (VPS) may be beneficial. Our study aimed to describe the cognitive, neuroimaging-MRI (cerebrospinal fluid-CSF volumetric analysis), and lumbar infusion test features of a cohort of cancer survivors (*n* = 36) with suspected post-RT NPH and identify which patients may benefit from a VPS. It was revealed that up to 81% of our cohort met the criteria for cognitive impairment. Additionally, we observed that the addition of a CSF volumetric analysis improved the identification of VPS responders (accuracy of 93%), thus enhancing the management and prognosis of long-term cancer survivors.

**Abstract:**

Background: We examined cognitive, brain MRI, and lumbar infusion test (LIT) features to identify predictors of response to ventriculoperitoneal shunting (VPS) in long-term cancer survivors with suspected normal pressure hydrocephalus (NPH) following cranial radiotherapy (RT). Methods: Patients who completed cranial RT at least 2 years before with clinically suspected NPH and an Evans’ index (EI) ≥ 0.30 underwent a cognitive and a cerebrospinal fluid (CSF) volumetric (MRI) analysis (*n* = 36). For those in whom VPS was placed (*n* = 14), we explored whether adding a CSF volumetric analysis to classical MRI and LIT (Tap Test) features would better identify VPS responders. Results: Nearly 80% exhibited cognitive impairment. The CSF volume at NPH diagnoses was significantly larger in the group of VPS responders (*p* = 0.04). The addition of CSF volume to NPH diagnoses increased accuracy to 93%, with a positive and negative predictive value of 91% and 100%, respectively. Conclusion: The addition of a quantitative MRI analysis of CSF volume to classical MRI and LIT NPH criteria, along with a high clinical suspicion of NPH, may help to identify VPS responders, thus improving the clinical management and prognosis of long-term survivors.

## 1. Introduction

Nowadays, cranial radiotherapy (RT), is still one of the mainstay treatments for brain tumour patients. Despite the poor prognosis of this population, some patients will achieve long-term overall survival. These include patients with low-grade or even high-grade primary brain tumours and patients with brain metastases with favourable clinical and molecular prognostic factors.

However, 50–90% of them will experience long-term cognitive deficits induced by cranial RT, impairing functional independence and quality of life [1,2,3,4,5,6,7,8]. Radiation-induced cognitive impairment is mainly characterized by deficits focused on attention, verbal and visual memory, visuospatial skills, and executive functions, and it can be disabling and permanent [9,10,11,12].

Typically, it has been considered that RT-induced neurotoxicity results from direct damage to neurons or glia or from disruption of the normal cerebral vasculature [13]. Radiologically, it is observed as cerebral atrophy, white matter (WM) damage, and ventricular dilatation [12,13,14,15,16,17]. The mechanisms of ventricular dilatation due to cranial RT are not yet fully understood. One of the postulated hypotheses is that cranial RT might induce the production of transforming growth factor β (TGF-β) leading to arachnoid granulation fibrosis and decreasing cerebrospinal fluid (CSF) drainage [17,18,19]. Ventricular dilatation is often disproportionate to the cerebral atrophy, and it manifests clinically like normal pressure hydrocephalus (NPH): insidiously progressive gait disturbance, urinary incontinence, and cognitive impairment.

Thiessen and DeAngelis in 1998 conducted a retrospective review of the potential benefit of ventriculoperitoneal shunting (VPS) in patients with RT-induced leukoencephalopathy (*n* = 30). They found that more than half of their patients experienced a favorable functional response after VPS, although only urinary incontinence achieved a statistically significant difference. Cognitive impairment showed minimal improvement, but it is important to note that only one third received formal neuropsychological testing [16]. Since then, only a few short series of patients or isolated clinical cases have been reported regarding hydrocephalus following cranial RT and the potential benefit of VPS, resulting in no consensus on its diagnosis and management [17,20,21].

In the absence of any standard criteria for NPH, a combination of clinical and neuroimaging features, such as Evans’ Index (EI, the ratio of the maximum width of the frontal horns to the maximum width of the inner table of the cranium) and disproportionately enlarged subarachnoid space hydrocephalus (DESH), are relevant to support diagnoses [22,23,24]. Currently, EI measurements greater than 0.30 are considered hydrocephalus; the first step and the quickest way to demonstrate ventriculomegaly. Moreover, it seems to be a robust MRI finding preceding the onset of symptoms in idiopathic NPH [25]. In addition, to support the important role that neuroimaging plays in the diagnosis of NPH, in 2010, the concept of DESH (tight high convexity and enlarged sylvian fissures with ventriculomegaly) was introduced in idiopathic NPH guidelines, demonstrating a high positive predictive value (77%) in identifying shunt responsive idiopathic NPH patients [26]. Furthermore, the CSF tap test or CSF Drainage Test, as well as Lumbar Infusion Test (LIT), are mainly used to support the diagnosis when there is a high clinical suspicion and no typical MRI features are found due to their high false-negative rates [23].

Despite the fact that these assertions have been widely established in idiopathic NPH, there are still no clinical guidelines for secondary NPH. Secondary NPH, such as post-RT NPH, encompasses a diverse group of acquired hydrocephalus, with subarachnoid hemorrhage and traumatic brain injury being the most common causes. Interestingly, differences in outcome between idiopathic and secondary NPH have been observed, with substantially better outcomes for secondary than for idiopathic NPH following VPS [27,28,29].

Therefore, identifying those cancer survivors with RT-induced cognitive impairment and NPH who may benefit from VPS is crucial. While some predictors of VPS responsiveness, including the presence of DESH or a positive tap test, have been described in idiopathic NPH, to the best of our knowledge, no reliable predictor of successful outcomes for shunting has been associated with secondary NPH.

Our study examines cognitive deficits, as well as neuroimaging and infusion test features, in a series of patients who have a previous history of cranial RT and progressive cognitive decline together with urinary incontinence and/or gait disturbance, in which post-RT NPH is suspected.

The aim of our study is to identify the clinical, neuroimaging, and infusion test characteristics of those patients with post-RT NPH who may benefit from VPS.

## 2. Materials and Methods

### 2.1. Patients

Medical records of patients with progressively worsening of one or more of the clinical triad symptoms of NPH (cognitive decline, urinary incontinence, and gait disturbance) who underwent cranial RT treatment at least 2 years before the study’s initiation were retrospectively reviewed. We decided to use 2 years as a minimum interval of time from RT, to avoid selecting patients who might exhibit reversible cognitive deficits following RT [9]. Patients included in the study were over 18 years old and underwent both an MRI and LIT between January 2014 and July 2022 at the Hospital Universitari de Bellvitge-Institut Català d’Oncologia, L’Hospitalet. Exclusion criteria included previous exposure to RT and previous or concurrent neurological disorders such as aphasia or marked visual compromise that may complicate cognitive evaluation. All patients were right-handed and fluent Spanish speakers. This study was approved by the Clinical Research Ethics Committee of the Hospital Universitari de Bellvitge (reference number PR330/21).

### 2.2. Neuropsychological Assessment

Patients were evaluated using a verbal memory test [Hopkins Verbal Learning Test-Revised (HVLT-R)] [30,31], a visuospatial abilities and visual memory test [Rey-Osterreith Complex Figure Test (ROCF) Copy and Delayed recall] [32], a verbal fluency test [Controlled Oral Word Association (COWA)] a processing-speed test [Trail Making Test (TMT) A-B] [33]. Raw cognitive test scores were compared with the validated Spanish normative values, corrected for age and education, and converted into z-scores. If inadequate completion was noted due to moderate cognitive compromise, the Mini-Mental State Examination (MMSE) was completed.

Cognitive impairment was defined as subjective memory complaints accompanied by an impairment in one or more cognitive domains that are greater than would be expected for the patient’s age and educational level (one test scored ≥2 standard deviations (SD) below the sample mean or two tests scored ≥1.5 SD below the sample means) [33,34] and/or a MMSE score below 27 when the neuropsychological assessment was poorly completed [35].

### 2.3. Lumbar Infusion Test and Data Acquisition

LIT is a method used to measure the resistance of CSF outflow (Rout). It consists of inserting a lumbar cannula into the CSF space while the patient is lying horizontally on the left side, and then the examiner determines the opening CSF pressure (P_op_). After that, through another lumbar cannula, following the constant infusion method, an artificial CSF (saline solution) is infused at a rate of 1.6 mL/min for about 20 min, until pressure reaches a plateau level (P_p_) [36]. Rout is calculated by P_p_ minus P_op_ divided by infusion rate (Rout = P_p_ − P_op/_infusion rate). The duration of the register is between 40–60 min. If the baseline pressure exceeds 50 mmHg or if the patient experiences headaches or neurological symptoms, the infusion test is interrupted. Following the infusion test, a CSF tap test is performed. It consists of removing 20–30 mL of CSF and after an interval of time (less than 72 h) evaluating improvement of gait, urinary incontinence, or cognitive symptoms [37]. Prior to performing the LIT, all patients were clinically evaluated using a score similar to that of Sorteberg et al. [38] (Table 1), according to our hospital protocol. Clinical improvement after VPS placement was defined as an improvement in one or more items of this score as evaluated by a neuro-oncologist.

### 2.4. MRI Data and Image Processing for Volumetric CSF Analysis

The MRI (1.5 Tesla) closer to the infusion test date was examined. Brain morphology was assessed, including: (a) EI (ratio of the maximum width of the frontal horns to the maximum width of the inner table of the cranium), for which measurements greater than 0.30 are considered hydrocephalus; (b) other features of DESH, that include tight high convexity or midline surfaces, an enlarged sylvian fissure associated with ventriculomegaly, and focal dilatation of sulci not attributable to atrophy [22,23,24,39].

We used the Fazekas scale to grade WM changes or leukoencephalopathy in both periventricular and deep WM areas [40]. Additionally, we performed a quantitative CSF volumetric analysis. First, if necessary, the remaining tumour and/or surgical cavity were identified and drawn in native space over the T1-weighted imaging for each patient using MRIcron (http://www.mccauslandcenter.sc.edu/mricro/mricron (accessed on 2 September 2019)). Then, a morphometric analysis was carried out using the SPM8 software package (Welcome Department of Imaging Neuroscience Group, London, UK) running on MATLAB (v7, Mathworks, Natick, MA, USA). Specifically, first, Unified Segmentation with medium regularization and cost function masking were applied to segment gray matter (GM), WM and CSF images and normalize the T1 image and the tumour mask for each patient into the Montreal Neurological Institute (MNI) space [41]. After segmentation, we calculated the volumes of GM, WM, and CSF. Next, we modulated the CSF normalized images by total intracranial volume (TIV) (GM + WM + CSF) to account for potential brain volume differences between patients.

All statistical analyses were conducted using SPSS 27.0 software (SPSS Inc., Chicago, IN, USA). We used U-Mann-Whitney, Chi-square, Student-t and log-rank tests to assess for group differences, depending on the nature of the variables, with a critical *p* threshold of 0.05. Additionally, a receiver operating characteristic (ROC) curve analysis was conducted to determine a cutoff of CSF volume with desirable levels of sensitivity and specificity to predict VPS responses.

## 3. Results

### 3.1. Demographic, Clinical, LIT and MRI Characteristics

A total of 50 patients (median time from cranial RT to study entry was 4 years, range 2–23 years) were screened: 36 patients (72%) had an EI ≥ 0.30 and 14 patients (28%) had an EI < 0.30 which were excluded from further analysis. All patients underwent a cytological examination of the CSF during LIT, which ruled out leptomeningeal carcinomatosis. Table 2 shows the characteristics of the entire cohort. Patients scored worse on gait disorder than on cognitive deficits or urinary incontinence. Almost half of our cohort received whole-brain RT and the other half partial-brain RT. Regarding MRI features, less than half of the patients exhibited DESH. All patients had a CSF opening pressure < 18 mmHg [22,27]. Mean Rout value was higher than 12 mmHg/mL/min (reference value for predicting shunt responsiveness in NPH patients) [42]. Twenty-two out of 36 patients (61.1%) had a Rout > 12 mmHg/mL/min, of which only 10 patients (10/36, 27.8%) also had a positive tap test.

### 3.2. Neuropsychological Assessment

Table 3 shows the results of the neuropsychological evaluation. Of the 36 patients included, 26 (72.2%) completed all the tests included in the neuropsychological battery. Six patients only completed the MMSE test with a median score of 25 (range 13–29) and the other 4 patients refused to do cognitive testing due to fatigue.

Overall, 26 out of 32 patients (81%) met the criteria for cognitive impairment, specially focused on executive functioning (Trail making test B) but also on processing speed (Trail making test A) and verbal fluency.

### 3.3. Ventriculoperitoneal Shunting

Patients with NPH suspicion who showed more than one of the clinical triad symptoms, an EI ≥ 0.30 and a pathological Rout value > 12 mmHg/mL/min, a criterion used by the neurosurgeons of our institution, were eligible for VPS placement. Twenty patients of our cohort met these criteria (20/36, 56%), but VPS was eventually ruled out in six patients due to either patient refusal (*n* = 4) or recurrence/disease progression (*n* = 2). As a result, a total of 14 out of 36 patients included (39%) underwent VPS placement.

**Figure 1:**
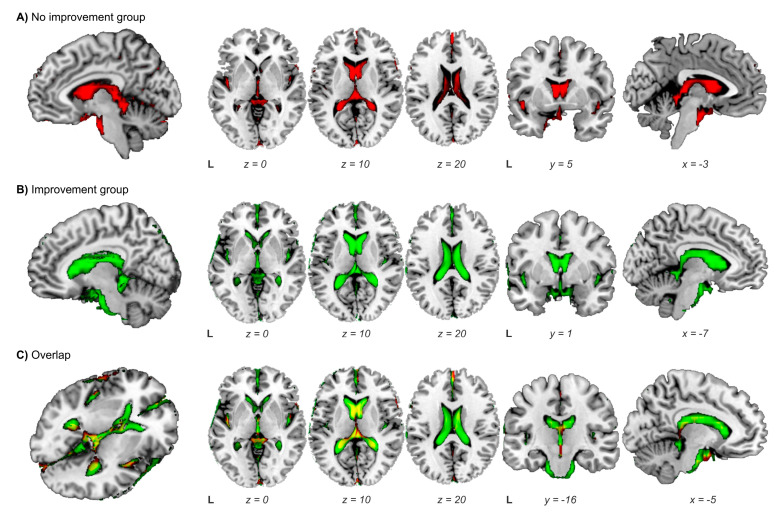
Representation on a standardized T1 template of the cerebrospinal fluid (CSF) volume of patients undergoing a ventriculoperitoneal shunt (VPS). CSF images were created from the sum of the individual CSF normalized images at NPH diagnoses. (**A**) In red: Group of patients with no clinical improvement post-VPS. (**B**) In green: Group of patients with clinical improvement post-VPS. (**C**) Overlap (in yellow) of the CSF volume of non-improvement (red) vs. improvement group (green). CSF volume at NPH diagnoses was higher in the improvement group (green) compared to the non-improvement group (red) (*p* = 0.04). L, left.

Overall, 71% of patients (10/14) showed clinical improvement after VPS (with a median time between VPS and clinical assessment of three months, range 1–6 months). There were no differences regarding demographic, clinical, and LIT features between those who improved or not following VPS. Only RT doses resulted in significant differences between groups. This is explained by the fact that in the non-improvement group, there was one patient who had received more than 60 Gy of RT (initially having received partial-brain RT and subsequently fractioned stereotactic radiosurgery due to a local recurrence). When we repeated the analysis without this outlier, RT doses resulted in no significant differences (*p* = 0.11). Concerning neuroimaging features, neither the EI value nor the presence of DESH showed significant differences between groups. In contrast, CSF volume at NPH diagnoses was significantly larger in the improvement group than in the non-improvement (*p* = 0.04) (See Figure 1). See Table 4 for characteristics.

Patients with post-RT NPH who did not undergo VPS, failed to show any clinical improvement compared to the VPS group (*p* < 0.001). In fact, during the 6-month follow-up evaluation, 14 out of 21 (67%) patients experienced a worsening of their NPH symptoms. One patient could not be evaluated because he died from pneumonia prior to the clinical follow-up.

### 3.4. Ventriculoperitoneal Shunting: Identification of Responders

**Figure 2:**
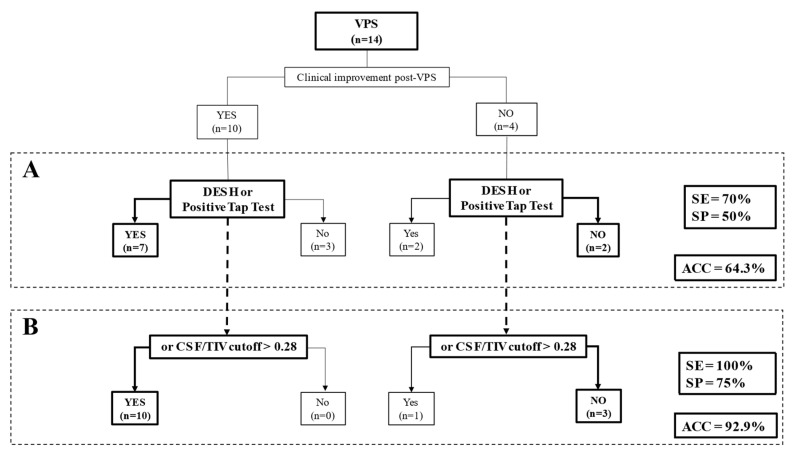
(**A**) Flow diagram of sensitivity (SE), specificity (SP) and classification accuracy (ACC) of considering the presence of disproportionate enlarged subarachnoid space hydrocephalus (DESH) or a positive tap test for clinical improvement after ventriculoperitoneal shunting (VPS) placement are shown. (**B**) Flow diagram of SE, SP and ACC of considering the presence of DESH or a positive tap test or cerebrospinal fluid (CSF)/total intracranial volume (TIV) cutoff > 0.28 for clinical improvement after VPS placement are shown.

We then explored the potential predictive factors of shunt responsiveness in our cohort. See Figure 2. Based on previous studies and idiopathic NPH guidelines, in which the presence of DESH or a positive tap test were considered supportive features for VPS [22,23,24,43]; we first analysed the predictive value of both in our cohort. We found that when we classified VPS patients based on these features, we could identify VPS responders with a positive predictive value (PPV) of 78%, a negative predictive value (NPV) of 40%, and a sensitivity and specificity of 70% and 50%, respectively. The classification accuracy was of 64.3% (Figure 2A).

Secondly, because CSF volume (CSF volume/TIV) resulted in the only significant differential factor between groups (clinical improvement post-VPS vs. no clinical improvement post-VPS) we added this variable to the analysis. After applying a ROC curve analysis to the variable CSF volume [area under the curve (AUC) = 0.88, *p* = 0.034], a cutoff of CSF volume of 0.280 mL was used. Thus, when we considered DESH or tap test together with a cutoff CSF volume of >0.280 mL, the identification of those patients who may benefit from VPS improved, with a PPV of 91% and NPV of 100%, and a sensitivity of 100% and a specificity of 75%. In that case, the classification accuracy increased to 92.9% (Figure 2B). Interestingly, we observed that when only CSF volume was taken into account, without considering the presence of DESH or tap test result, we achieved the same accuracy (92.9%).

Finally, it is also worth pointing out that 29% of the patients (4/14) experienced complications after VPS placement. Three patients developed subdural collections (one patient also had VPS infection); two of whom required neurosurgical drainage and revision of the VPS. The other patient required a VPS repositioning due to poor abdominal position. However, all complications were successfully resolved.

## 4. Discussion

The present study describes the cognitive, neuroimaging, and CSF characteristics of the largest published series of cancer patients with suspected post-RT NPH, highlighting the use of CSF volumetrics to identify potential VPS responders.

Our work revealed that up to 81% (*n* = 26/32) of our cohort met the criteria for cognitive impairment, with more pronounced deficits focused on executive functioning, accompanied in most cases by severe leukoencephalopathy (median time since cranial RT of 4 years). These findings are consistent with previous studies of radiation-induced cognitive toxicity, including the retrospective study by Thiessen and DeAngelis [7,8,11,12,20]. The latter is by far the first study to report the cognitive profile of patients with RT-induced hydrocephalus; however, only one third of the 30 patients included underwent a complete neuropsychological battery, and the median time since cranial RT or the grade of leukoencephalopathy associated were not reported.

Considering that both cranial RT and NPH have a detrimental effect on WM integrity, especially in the frontal-subcortical structures [9,11,12,44,45,46,47,48], it can be challenging to distinguish between radiation-induced cognitive changes and cognitive deficits related to NPH. Previous small, but interesting diffusion tensor imaging studies showed that subcortical frontal WM fasciculi involved in executive functioning were compromised in idiopathic NPH compared to healthy controls, Alzheimer’s disease, or Parkinson’s disease. It has been speculated that the presence of hydrocephalus could, through a mechanical effect, compress and disrupt these subcortical WM structures [47,48].

As found by Thiessen and DeAngelis [16], around 70% of our patients obtained a favourable functional response after VPS. Neither classical neuroimaging nor LIT taken as isolated features were useful enough to differentiate VPS responders from non-responders in our cohort. Interestingly, in the same line, previous studies as well as idiopathic NPH guidelines have concluded that LIT features, including CSF dynamics and tap test, may have false negative results. In fact, in idiopathic NPH, VPS placement is recommended for patients with a high clinical suspicion, accompanied by ventriculomegaly, who exhibit features of DESH, regardless of tap test results [22,23,24,38,44].

Bearing this in mind and considering that in our cohort VPS responders had a significantly higher CSF volume than non-responders (*p* = 0.04), we explored whether the addition of a quantitative MRI analysis of CSF volume to the classical NPH MRI criteria and tap test result might improve the identification of VPS responders. Specifically, we showed that CSF volumetric analysis, as add-on variable but also by itself, achieves a 92.9% accuracy in differentiating VPS responders from non-responders. Interestingly and in line with our results, several studies focusing on quantitative MRI CSF volumetric analysis in idiopathic NPH patients have been published in the past decade [49,50,51]. They agreed that the assessment of CSF volumetric could differentiate both NPH patients from other pathologies and between VPS responders from non-responders.

Our study presents some limitations. Firstly, it was a single-centre study; thus, multicentre and randomized studies are required to validate the results. Secondly, due to its retrospective nature, longitudinal cognitive testing was not performed following VPS, thus hampering the assessment of objective cognitive improvement after VPS. Additionally, the small number of NPH patients undergoing VPS placement (*n* = 14) may have impeded the achievement of significant differences between responders and non-responders, limiting the generalizability of the results obtained. Additionally, while the number of patients who developed complications after VPS appeared considerable (29%), all of them were successfully managed with a favourable outcome. 

## 5. Conclusions

In conclusion, our study has shown that the addition of a quantitative CSF MRI analysis in the study of patients with clinical suspicion of post-RT NPH could aid in identifying those who may benefit from VPS, thus improving the clinical management and prognosis of long-term cancer survivors. However, larger multicentre studies are needed to confirm the usefulness, accuracy, and applicability of this approach.

## Figures and Tables

**Table 1:** Normal pressure hydrocephalus score: Gait, dementia, and urinary incontinence.

Gait		Dementia		Urinary Incontinence	
Wheelchair, bedridden	5	Institutionalized due to dementia. Patient may no longer survive without assistance	5	Permanent urinary catheter	5
Support of another person to walk	4	Severe dementia. Partial loss of independence	4	Constant incontinence	4
Support of a cane to walk	3	Moderate decreased memory and behaviour but independent patient	3	Occasional incontinence	3
Abnormal, but walking possible without support	2	Subjective feeling of decreased memory	2	Rare incontinence	2
Normal	1	None	1	None	1

**Table 2:** Clinical, treatment, magnetic resonance imaging and infusion test features (*n* = 36).

Age, years at diagnosis (mean ± SD)	56 ± 13.52
Years of education (mean ± SD)	13.33 ± 2.73
Level of education, n (%)	
Primary School	2 (5.6)
Lower Secondary School	26 (72.2)
Upper Secondary School	5 (13.9)
College	3 (8.3)
Gender, n (%)	
Male	18 (50)
Female	18 (50)
NPH clinical score, median (range)	
Gait abnormalities	4 (1–5)
Cognitive deficits	2 (1–3)
Urinary incontinence	2 (1–5)
Brain Tumour diagnoses, n (%)	
Primary	20 (55.6)
Metastases	8 (22.2)
Prophylactic cranial irradiation	8 (22.2)
Surgery, n (%)	23 (63.9)
Extent of surgical resection	
Gross total resection	12 (52.2)
Partial resection	11 (47.8)
RT type, n (%)	
Whole-brain RT	16 (44.4)
Partial-brain RT	18 (50)
Combination of cranial RT techniques	2 (5.6) ^a^
Total cranial RT dose Gy, median (range)	50 (32–95)
Time between cranial RT and hydrocephalus suspicion (years), median (range)	4 (2–19)
MRI features	
Fazekas scale, median (range)	3 (1–3)
EI ratio (mean ± SD)	0.33 ± 0.03
Presence of DESH, n (%)	16 (44.4)
CSF volume/TIV, mL (mean ± SD) ^b^	0.37 ± 0.06
LIT features:	
Time between cranial RT and LIT (years), median (range)	4 (2–20)
CSF opening pressure, mmHg (mean ± SD)	10.37 ± 3.63
ROUT, mmHg/ml/min (mean ± SD)	13.20 ± 4.18
Positive Tap Test, n (%)	15 (41.7)

CSF, cerebrospinal fluid; DESH, disproportionately enlarged subarachnoid space hydrocephalus; EI, Evans’ Index; LIT, lumbar infusion test; MRI, magnetic resonance imaging; NPH, normal pressure hydrocephalus; Rout = P_p_ − P_op/_infusion rate; RT, radiotherapy; SD, standard deviation; TIV, total intracranial volume. ^a^ 1 patient received whole-brain RT and subsequently intensity-modulated radiation therapy due to an atypical radiation-induced meningioma. 1 patient received partial-brain RT and subsequently fractionated stereotactic radiosurgery due to a local recurrence. ^b^ 3 patients were not included due to MRI segmentation errors.

**Table 3:** Neuropsychological results (*n* = 32).

Time between cranial RT and neuropsychological tests (years), median (range)	4 (2–23)
Cognitive impairment, n (%)	26 (81.3)
≥1 cognitive domain impaired	21 (80.8)
MMSE score < 27	5 (19.2)
Phonemic fluency, mean ± SD	
COWA	−1.02 ± 0.79
Visuospatial abilities, mean ± SD	
ROCF First Copy	1.10 ± 1.53
Visual memory, mean ± SD	
ROCF Delayed Copy	−0.08 ± 0.77
Verbal memory, mean ± SD	
HVLT total recall	−0.85 ± 1.01
HVLT delayed recall	−0.56 ± 1.33
HVLT delayed recognition	0.28 ± 1.64
Processing speed/executive functions, mean ± SD	
Trail Making Test A	−1.33 ± 0.83
Trail Making Test B	−1.51 ± 0.81

COWA, Controlled Oral Word Association Test; HVLT, Hopkins Verbal Learning Test; MMSE, Minimental State Examination; ROCF, Rey-Osterrieth Complex Figure; RT, radiotherapy; SD, standard deviation.

**Table 4:** Clinical, infusion test and magnetic resonance imaging features of patients with ventriculoperitoneal shunting (*n* = 14).

	Clinical Improvement Post-VPS	No Clinical Improvement Post-VPS	*p* Value
	(*n* = 10)	(*n* = 4)	
Age, years at diagnosis (mean ± SD)	57.60 ± 11.57	54.75 ± 16.70	0.72
Gender, n (%)			1.00
Male	5 (50)	2 (50)	
Female	5 (50)	2 (50)	
NPH clinical score, median (range)			
Gait abnormalities	4 (3–5)	4 (4–5)	0.17
Cognitive deficits	2 (1–2)	2 (2–3)	0.23
Urinary incontinence	2 (1–4)	3 (3–4)	0.16
Brain Tumour diagnoses, n (%)			0.11
Primary	3 (30)	4 (100)	
Metastases	4 (40)	0 (0)	
Prophylactic cranial irradiation	3 (30)	0 (0)	
Surgery, n (%)	5 (50)	4 (100)	0.22
Extent of surgical resection			0.42
Gross total resection	1 (25)	2 (50)	
Partial resection	4 (75)	2 (50)	
Total cranial RT dose Gy, median (range)	45 (25–60)	60 (60–95) ^a^	**0.02**
MRI features			
EI (mean ± SD)	0.34 ± 0.04	0.37 ± 0.07	0.35
DESH, n (%)	6 (60)	1 (25)	0.56
CSF volume / TIV, mL (mean ± SD)	0.39 ± 0.08	0.30 ± 0.04	**0.04**
LIT features			
ROUT, mmHg/mL/min (mean ± SD)	17.32 ± 2.10	16.75 ± 1.72	0.62
Positive Tap Test, n (%)	5 (50)	1 (25)	0.58
Time between cranial RT and VPS (years) median (range)	4 (2–20)	8 (4–11)	0.33

CSF, cerebrospinal fluid; DESH, disproportionate enlarged subarachnoid space hydrocephalus; EI, Evans’ index; LIT, lumbar infusion test; MRI, magnetic resonance imaging; NPH, normal pressure hydrocephalus; ROUT, Rout = P_p_ − P_op/_infusion rate; RT, radiotherapy; SD, standard deviation; TIV, total intracranial volume; VPS, ventriculoperitoneal shunting. ^a^ 1 patient received partial-brain RT and subsequently fractionated stereotactic radiosurgery due to a local recurrence.

## Data Availability

Anonymized data for our analyses presented in this report are available upon request from the corresponding author.
